# The role of the plantar fascia, flexor hallucis longus, and fibro-osseous tunnel in functional hallux rigidus

**DOI:** 10.1007/s00402-026-06489-9

**Published:** 2026-09-27

**Authors:** Miqi Wang, Alexandra Hunter Aitchison, Adrienne M. Orriols, Ryan M. Ridenour, Mark E. Easley, Samuel B. Adams

**Affiliations:** 1 University of Florida, Gainesville, USA; 2 Duke University, Durham, USA; 3 Greater Pittsburgh Orthopaedic Associates, Pittsburgh, USA

**Keywords:** Functional hallux rigidus, Plantar fascia, Flexor hallucis longus, Fibro-osseous tunnel

## Abstract

**Background:**

Functional hallux rigidus is characterized by limited first metatarsophalangeal (MTP) dorsiflexion during weight bearing conditions, but normal motion otherwise. This phenomenon alters the biomechanics of the foot, potentially leading to first MTP pain and trauma to the MTP cartilage. The purpose of this study is to assess the role of the flexor hallucis longus (FHL), plantar fascia (PF), and fibro-osseous tunnel in functional hallux rigidus.

**Method:**

Fourteen cadaveric lower limbs were analyzed. The Dananberg technique was used to dorsiflex the great toe and radiographs were taken after each of the following manipulations, measuring the dorsiflexion angle of the first MTP. The plantar fascia was transected with a Z-cut, shortened approximately 10 mm, then repaired. The fibro-osseous tunnel was released. The FHL was transected with a Z-cut then shortened approximately 7 mm.

**Results:**

Average passive dorsiflexion of the first MTP is 23.8° (95% CI 19.7–27.9) prior to manipulation. Shortening the plantar fascia and FHL did not decrease the MTP dorsiflexion significantly beyond baseline (p>0.05). Lengthening the plantar fascia increased the dorsiflexion by 6.7° (28.1% increase) and lengthening the FHL increased dorsiflexion by 7.0° (29.4% increase). The difference between the two methods was not significant (p>0.05). Releasing the fibro-osseous tunnel alone increased the dorsiflexion 3.4° (14.4% increase) and was not significantly different from PF or FHL lengthening.

**Conclusion:**

Functional hallux rigidus causes limitation of first MTP dorsiflexion when the first metatarsal head is prevented from plantarflexing. There is no difference between the contribution of the FHL versus the plantar fascia to functional hallux rigidus. The true etiology may be multifactorial and involve other structures such as the flexor hallucis brevis, first metatarsal, and gastrocnemius/soleus/achilles complex. As such, no clear surgical intervention can be recommended to improve functional hallux rigidus except in cases with clear-cut causes of stenosis.

## Introduction

Functional hallux rigidus has been discussed in the literature as early as the 1950s [1–4]. It is defined as having limited first metatarsophalangeal (MTP) dorsiflexion when the foot is weightbearing. With open chain motion, the first metatarsal can plantarflex, allowing the proximal phalanx to dorsiflex normally. However, when the metatarsal plantarflexion is blocked, such as during ambulation, phalangeal dorsiflexion is limited. Clinically, this can be assessed using the Dananberg technique: the ankle is held in neutral flexion and the examiner blocks the metatarsal head from plantarflexing while the great toe is dorsiflexed [5]. Functional hallux rigidus is distinct from structural hallux rigidus in that there are no arthritic changes in the MTP to cause a mechanical block to motion. While the etiology of structural hallux rigidus is multifactorial, it has been hypothesized that functional hallux rigidus may be a factor [4, 6, 7].

Bøjsen-Moller and Lamoreaux measured students’ gait using light emitting diodes and found that maximal metatarsophalangeal dorsiflexion during pushoff was 50–60° for barefoot walking, 45–50° for soft shoe walking, and 25–30° for stiff shoe walking [8]. The Coughlin and Shurnas classification of hallux rigidus equates 40–60° of first MTP dorsiflexion as 10–20% loss of normal motion [9]. As such, it is accepted that 60° of dorsiflexion at the first MTP is optimal for a normal toe-off phase, indicating that any limitation in this movement could markedly disrupt normal gait. Potential sequelae are numerous. With abnormal flexibility of the first MTP, the joint “rolls” instead of gliding [7], causing it to pivot at an eccentric center of rotation [4]. This can cause pain, cartilage injury, and possible eventual progression to structural hallux rigidus (Michelson and Dunn, Maceira). If the medial column becomes stiff and painful, patients may develop reactive cavovarus and cause transfer metatarsalgia, lateral overload, and further downstream injuries.

Functional hallux rigidus can affect patients of all ages. In the *Gould* study, the nine patients treated had an average age of 33 and ranged from 14 to 68 years. His patient group consisted almost exclusively of female patients [2]. It is unclear how the process changes as patients age and activity level decreases.

The etiology of functional hallux rigidus remains unclear despite its 70 years of discourse. Multiple causes have been proposed, but this study will focus on the plantar fascia (PF) and flexor hallucis longus (FHL) given the frequency with which they are discussed in the literature. The plantar fascia is the static contributor to hallux plantarflexion while the FHL and, to a lesser degree, the flexor digitorum longus, are the active contributors [10].

The plantar fascia originates from the medial tubercle of the calcaneus and extends into the forefoot forming superficial longitudinal tracts [11, 12]. A pair of sagittal septae surrounds each MTP and insert on either side of the flexor tendons. The windlass mechanism maintains the longitudinal arch of the foot through the plantar fascia. As the plantar fascia tightens, the arch rises, and vice versa. The first MTP adds a layer of complexity into the windlass mechanism, creating a dynamic truss [13, 14]. Dorsiflexion of the great toe increases tension in the plantar fascia and increases the height of the arch. The converse is true as well, which means that an increase in plantar fascia tension will limit dorsiflexion of the great toe.

The FHL originates from the posterior aspect of the fibula and inserts onto the plantar aspect of the distal phalanx of the great toe. Its tendon runs posteriorly to the talus, between the medial and lateral tubercles of the posterior process [15, 16]. It continues medially, inferior to the sustentaculum tali of the calcaneus. In these regions, it is bound to the bone by a thick tendon sheath, forming the fibro-osseous tunnel. Due to the restrictive structure of the tunnel, it can be a site of stenosis for the FHL tendon. The presence of a nodule or ganglion on the tendon proximal to the tunnel can cause triggering, similar to trigger finger. The presence of tenosynovitis can cause crepitus and pain with excursion of the tendon within the tunnel. The presence of the low-lying FHL muscle belly can limit full excursion of the tendon, causing a “stopper bottle phenomenon” in which great toe dorsiflexion is limited with the ankle in dorsiflexion but not in plantarflexion [15, 17]. Surgical release of the fibro-osseous tunnel has been shown to improve the excursing of the FHL and improve pain due to functional hallux rigidus [18].

The goal of this study was to elucidate the anatomic etiology of functional hallux rigidus. While there are other possible causes of functional hallux rigidus described in the literature, we chose to focus on the plantar fascia and FHL, as these are the most cited and most anatomically relevant structures. Additionally, we sought to evaluate fibro-osseous tunnel release as a treatment for functional hallux rigidus.

## Methods

Fourteen fresh-frozen cadaveric lower limbs disarticulated at the hip were analyzed. The specimens were assessed for first MTP pathology, such as hallux valgus and structural hallux rigidus (including osteoarthritic changes). Limbs that have had previous first ray surgery were also excluded. Limbs whose ankles could not be dorsiflexed to neutral for evaluation via the Dananberg technique were excluded. All procedures and measurements were performed by the same Foot and Ankle fellowship-trained physician to minimize inconsistencies in assessment and surgical technique.

**Fig. 1:**
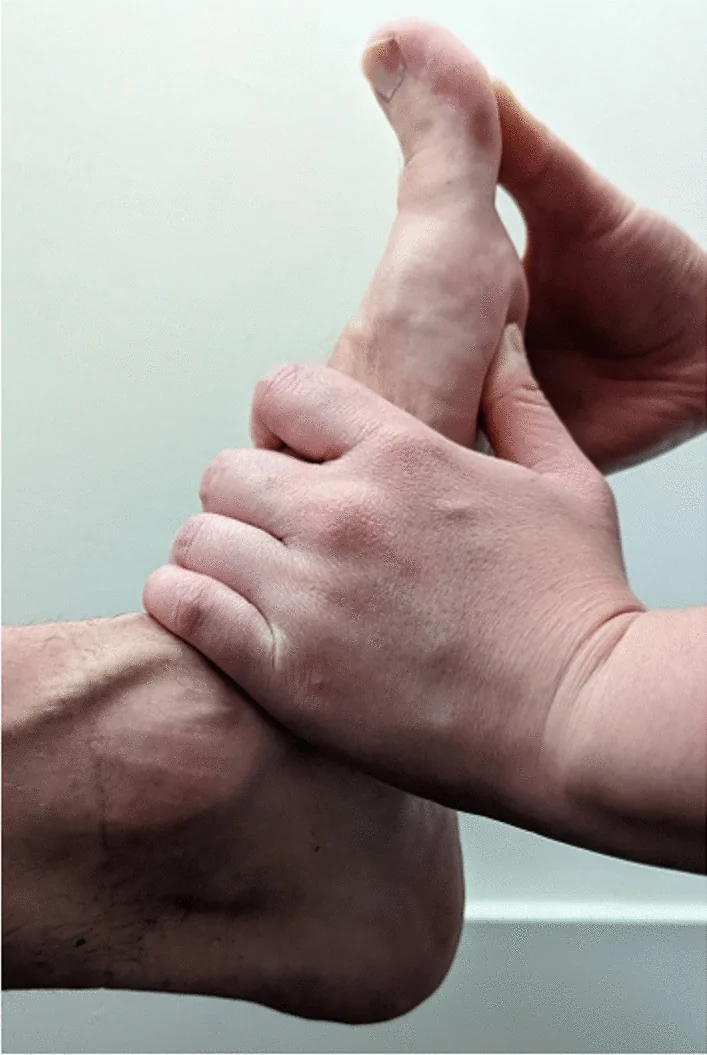
Dananberg technique of assessing functional hallux rigidus. The examiner’s thumb is used to stabilize the first metatarsal head plantarly to prevent plantarflexion while the other thumb is used to dorsiflex the great toe. The ankle is held in neutral and the dorsiflexion angle of the first metatarsophalangeal joint is assessed

First MTP dorsiflexion with the metatarsal head stabilized was assessed using the Dananberg technique. The examiner’s thumb was used to stabilize the first metatarsal head plantarly and prevent plantarflexion to simulate the closed-chain kinetics of weight bearing (Fig. 1). The same hand was used to hold the ankle neutrally aligned to the leg. The other hand was used to maximally dorsiflex the great toe. Lateral radiographs of the feet were obtained via fluoroscopy with the first MTP held in this position so that accurate measurements could be obtained.

.

**Fig. 2:**
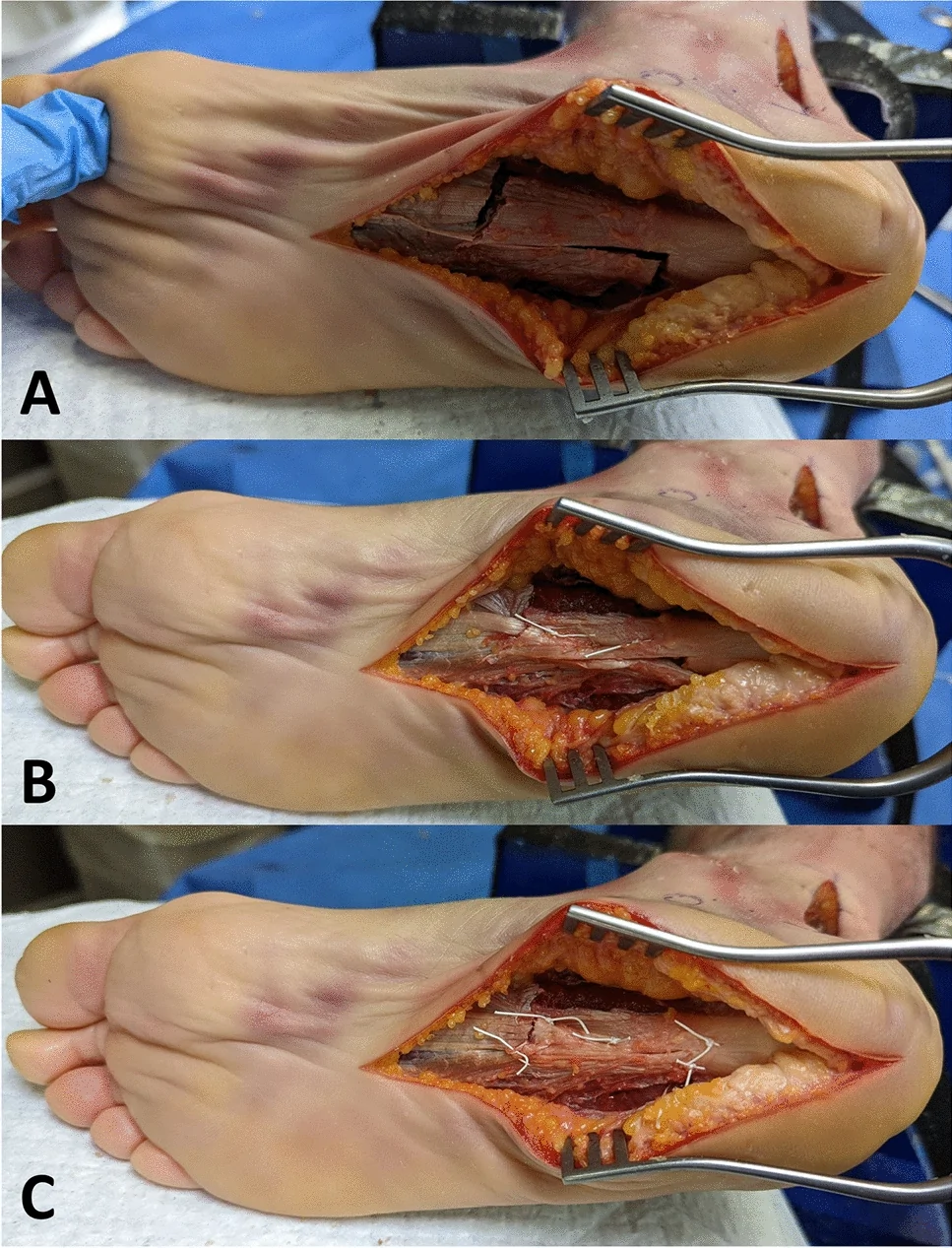
The plantar fascia is z-lengthened without repair (A), shortened 10 mm (B), and repaired at its original length (C)

The plantar foot was approached through a midline longitudinal incision spanning the midfoot and plantar heel. Soft tissue was sharply dissected until the plantar fascia was visualized and freed from surrounding tissue. The central band of the plantar fascia was then transected with a Z-cut (Fig. 2A) at the calcaneal insertion and allowed to lengthen freely. First MTP dorsiflexion was measured on lateral radiographs. The plantar fascia was then shortened approximately 10 mm (Fig. 2B) and first MTP dorsiflexion was measured again. Finally, the plantar fascia was repaired at its original length (Fig. 2C).

**Fig. 3:**
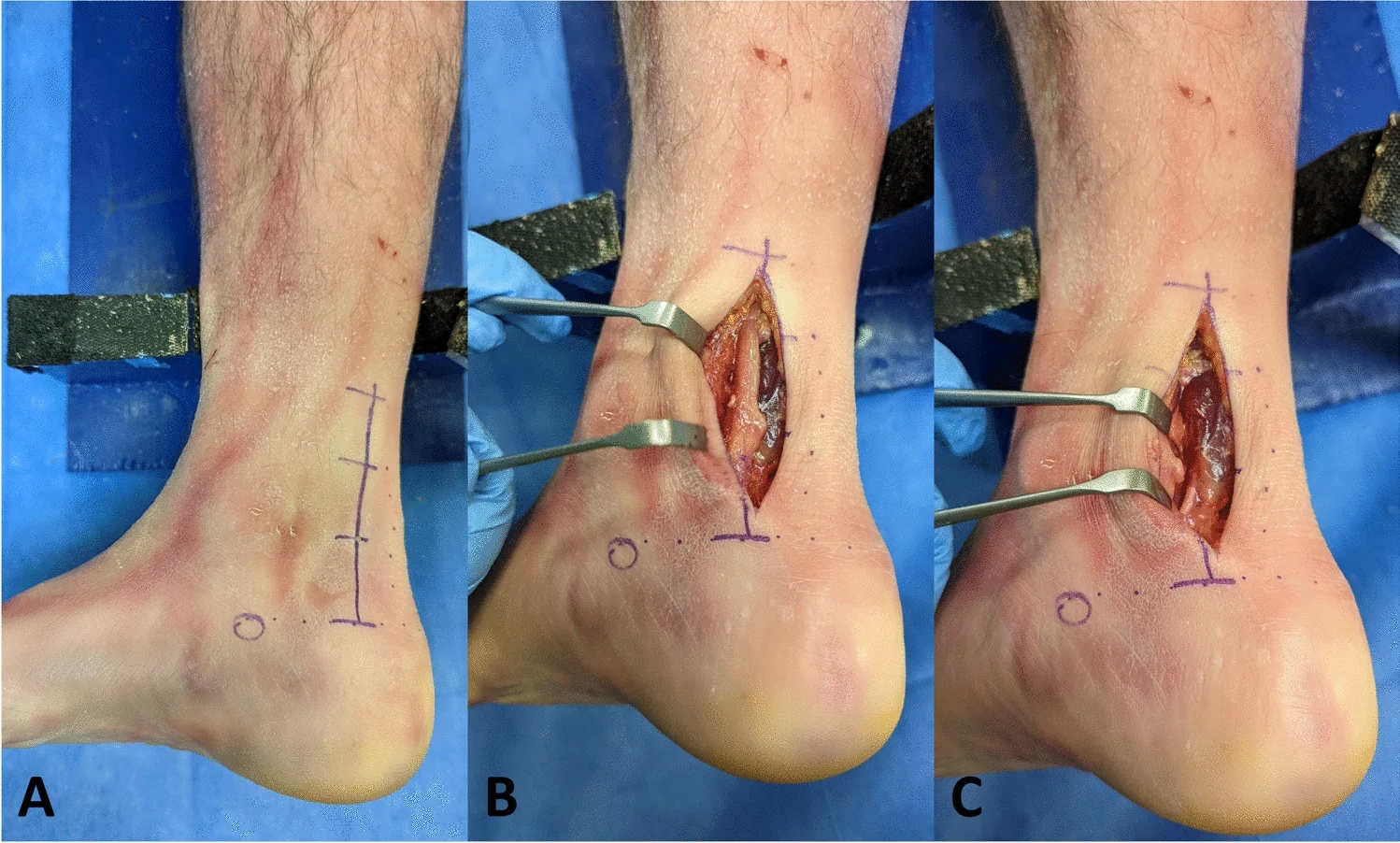
The flexor hallucis longus tendon was approached through a 4 cm incision made 1 cm anterior to the medial border of the Achilles tendon, terminating at the level of the sustentaculum tali (A). The neurovascular bundle was identified (B) and retracted anteriorly to expose the flexor hallucis longus tendon and muscle belly (C)

**Fig. 4:**
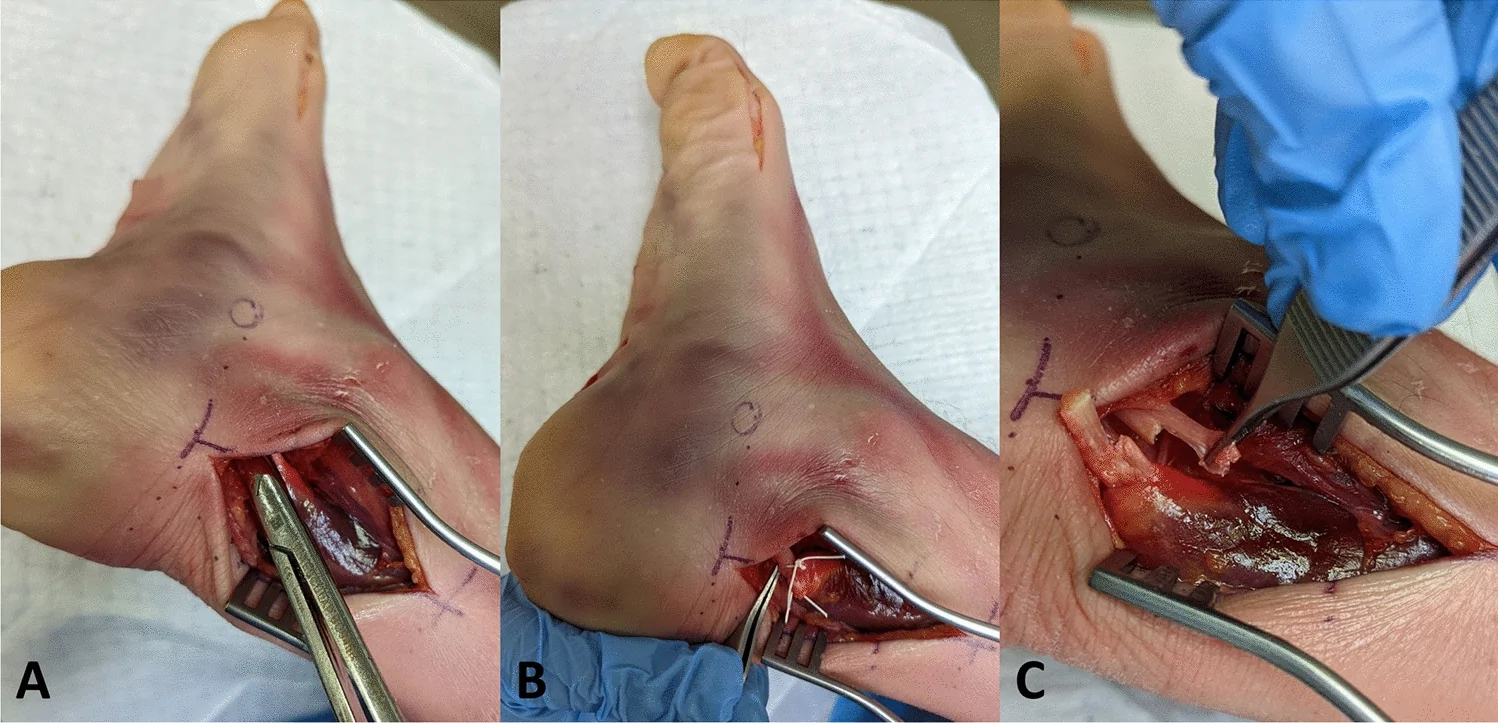
The flexor hallucis longus tendon was traced distally to identify the fibro-osseous tunnel, then released with Metzenbaum scissors (A). The flexor hallucis longus tendon was then shortened by 7 mm by suturing it in a plicated position (B), then transected with a z-cut (C)

We then turned our attention to the medial ankle. We used a posteromedial approach to the FHL. A 4 cm incision was made 1 cm anterior to the medial border of the Achilles tendon, terminating at the level of the sustentaculum tali (Fig. 3A). Soft tissue was dissected until the neurovascular bundle was identified (Fig. 3B). The bundle was carefully retracted anteriorly, exposing the FHL (Fig. 3C). The fibro-osseous tunnel was identified and released using Metzenbaum scissors and first MTP dorsiflexion was measured (Fig. 4A). The FHL was then shortened approximately 7 mm by suturing it in a redundant position (Fig. 4B). First MTP dorsiflexion was measured. Finally, the FHL was transected with a Z-cut (Fig. 4C), allowed to freely lengthen, and first MTP dorsiflexion was measured.

**Fig. 5:**
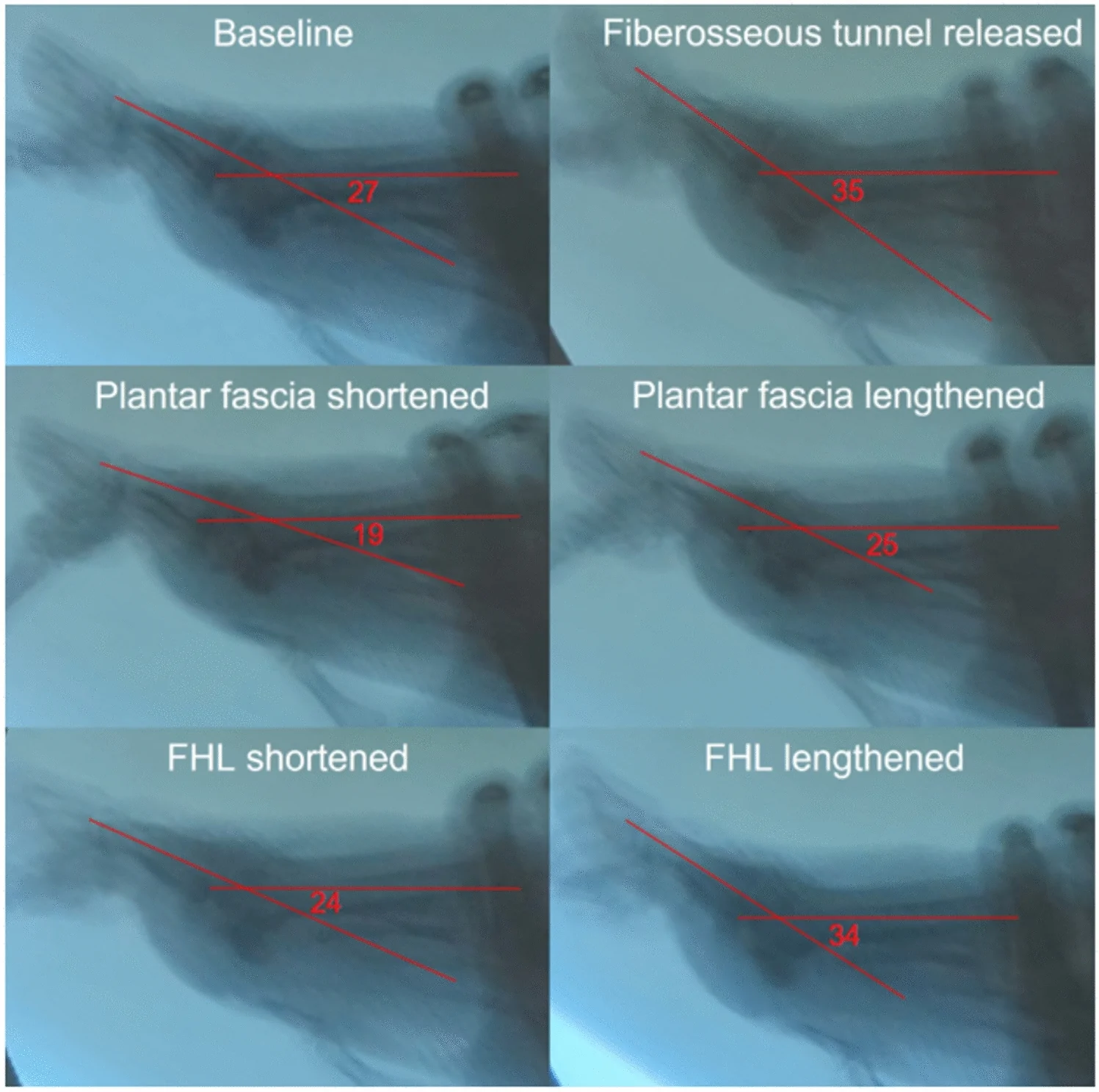
The Dananberg technique was used to assess first metatarsophalangeal dorsiflexion with the metatarsal head stabilized. Lateral radiographs were used to measure the changes in dorsiflexion at baseline, with the fibro-osseous tunnel released, with the plantar fascia shortened and lengthened, and with the flexor hallucis longus shortened and lengthened

First MTP dorsiflexion was measured by drawing the Cobb angle of the first metatarsal shaft and the first proximal phalanx shaft (Fig. 5). The change in dorsiflexion was found by subtracting the measured angle with the initial dorsiflexion achieved prior to fascial or tendon manipulation. To ascertain that the plantar fascia repair restored the first MTP dorsiflexion, the angle after PF repair was also compared against the initial angle. Confidence interval (95% CI) was calculated for the dorsiflexion measurements. Shapiro-Wilks test showed normal distribution of results. Analysis of variance (ANOVA) was used to assess the differences between the groups. We selected p = 0.05 for statistical significance. A subset of 21 measurements were performed by two authors to assess for interrater reliability via Pearson’s correlation coefficient.

## Results

The average age of the specimens was 79.7 years (range 60–95 years). The average baseline dorsiflexion was 23.8° (95% CI 19.7–27.9). Average dorsiflexion with the plantar fascia repaired at its original length was 19.8° (p = 0.26), indicating the repair was sufficient to restore baseline dorsiflexion. The average dorsiflexion with the PF lengthened is 28.8° (95% CI 22.6–34.9), shortened is 22.6° (95% CI 15.7–29.5). The average dorsiflexion with the FHL lengthened is 30.8° (95% CI24.3–37.3), shortened is 25.4° (95% CI 19.9–30.9). The average dorsiflexion with the fibro-osseous tunnel released is 27.3° (95% CI 20.6–34.1) (Table 1).

**Table 1:**
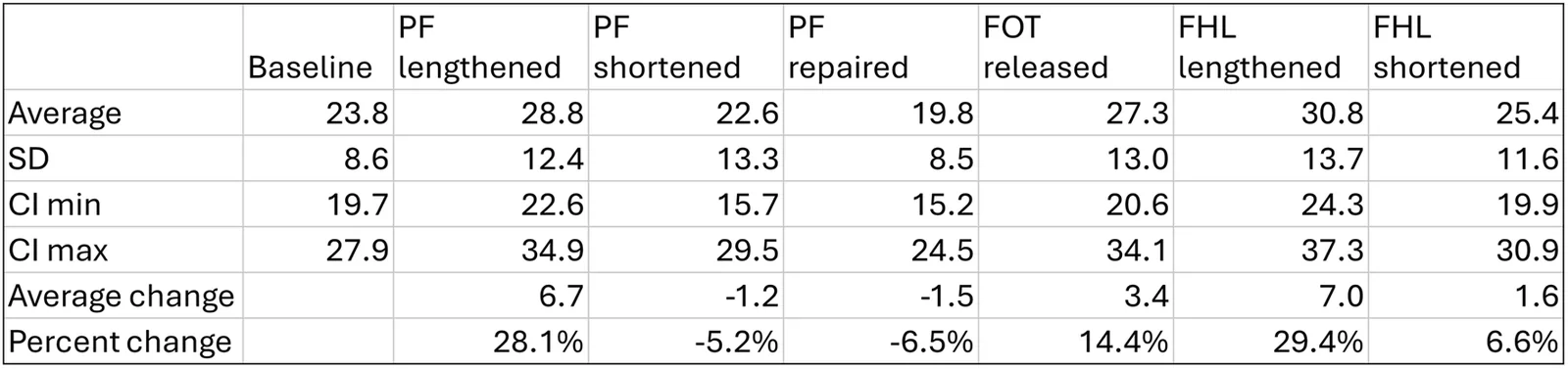
..

The average change in dorsiflexion with the plantar fascia lengthened was 6.7° (28.1% increase). The average change in dorsiflexion with the FHL lengthened was 7.0° (29.4% increase). The average change in dorsiflexion with the fibro-osseous tunnel released was 3.4° (14.4% increase). While these results trended towards increased dorsiflexion, ANOVA showed p = 0.30, indicating no statistical significance between any group.

There was no difference between the increase in dorsiflexion with the FHL lengthened versus PF lengthened. There is no difference in the increase in dorsiflexion between releasing the fibro-osseus tunnel and lengthening the FHL or lengthening the plantar fascia.

Shortening the PF resulted in a dorsiflexion change of − 1.2° (5.2% decrease) and shortening the FHL resulted in a change of 1.6° (6.6% increase). There was no difference in change in dorsiflexion with the FHL shortened versus PF shortened.

Due to the limitations of the dissection, the fibro-osseous tunnel release was only able to be performed after the PF manipulation and the FHL manipulation was only able to be performed after the fibro-osseous tunnel release. Using the post PF repair dorsiflexion as a baseline, fibro-osseous tunnel release alone results in a 24.2% increase in dorsiflexion. If the post-fibro-osseous tunnel release angle is considered the new baseline, then lengthening the FHL would result in 8.2% increase in dorsiflexion and shortening the FHL would result in a 10.7% decrease in dorsiflexion, none of which are significant (Table 2).

**Table 2:**
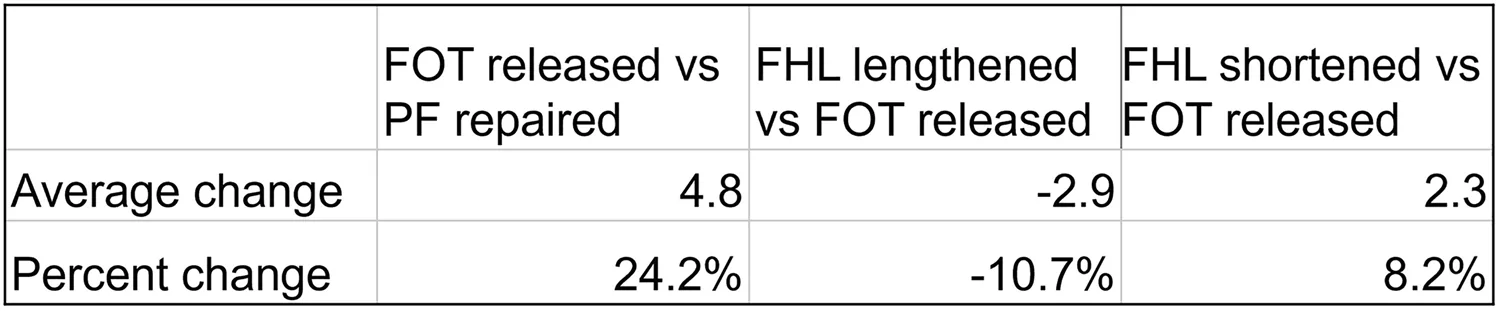
..

An assessment of a subgroup showed interrater reliability to 0.81, suggesting good reliability. As such, the measurements from a single foot and ankle fellowship trained surgeon were used to maximize consistency.

## Discussion

Lengthening both the plantar fascia and the FHL result in a non-significant trend towards increased dorsiflexion of the first MTP. There is no significant difference between the change in dorsiflexion between the two interventions found in this study. Similarly, there is no difference between the change in dorsiflexion when either plantar fascia or FHL are shortened. This suggests that both the plantar fascia and FHL are similarly implicated, or not implicated, in functional hallux rigidus. This would also explain why the literature remains conflicted about its pathophysiology.

Conversely, shortening the plantar fascia and the FHL do not significantly decrease the first MTP dorsiflexion. This is a bit of a surprising result, as we would expect a linear relationship between the implicated anatomy and the degree of dorsiflexion limitation. Perhaps the effect of plantar structural tightness on range of motion is more binary: first MTP dorsiflexion limitation either is or is not present and further shortening of these structures does not decrease range of motion. Kirane et al. show that restricting FHL excursion to as little as 2 mm beyond neutral leads to increased compression forces within the first MTP and dorsal MTP impingement [4], which is similar to the effects of early structural hallux rigidus. As such, if the specimen already had shortened or rigid FHLs and plantar fasciae due to age or preservation, additional shortening may not change MTP range of motion. In contrast, releasing the structures allowed infinite lengthening, which may explain the magnitude of change in dorsiflexion.

While the difference in range of motion change is statistically insignificant, this could be a result of the study being underpowered to differentiate between small changes. Shortening PF and FHL produce a decrease in dorsiflexion of almost 30% from baseline. There is no minimally clinically important difference established for first MTP range of motion, but the closest comparison would be the Coughlin Clinical-Radiographic System for Grading Hallux Rigidus [9]. A loss of 10–20% of dorsiflexion compared to the contralateral, unaffected side is associated with normal radiographic findings but clinical findings of stiffness and loss of motion (grade 0). A loss of 20–50% is associated with dorsal osteophyte formation on radiograph and mild/occasional pain and stiffness, pain with end range of motion (grade 1). By definition, grade 0 describes functional hallux rigidus and grades 1–4 describe structural hallux rigidus. As such, increasing dorsiflexion by more than 20% may restore a foot with functional hallux rigidus to baseline.

Similarly, if the post fibro-osseous tunnel release dorsiflexion is to be used as the baseline for comparison for FHL manipulation, then shortening the FHL results in a 10% decrease in dorsiflexion. This is in line with a Coughlin grade 0 describing stiffness and functional hallux rigidus.

Although releasing the fibro-osseous tunnel does not alter the length of the plantar fascia or FHL, it may enhance the excursion of the FHL. Of note, none of the specimens had FHL nodules or severely low-lying muscle bellies that would clearly affect FHL excursion within the tunnel. However, in patients who do have structural limitations or localized stenosis, releasing the fibro-osseous tunnel could result in improved dorsiflexion at the first MTP joint.

If the post PF repair dorsiflexion is to be used as the baseline for comparison for fibro-osseous tunnel release, then the release results in a 24% increase in dorsiflexion. While not statistically significant, this implies that fibro-osseous tunnel alone may be sufficient to restore Coughlin grade.

Nonoperative treatment of functional hallux rigidus as described by Michelson et al. is a viable and safe treatment option for functional hallux rigidus [19]. They describe elevating the hallux 1 cm to pretension the FHL, then maximally dorsiflexing the ankle to stretch the tendon along its entire length. While their study focuses on the FHL as the source of pain and the target of intervention, our study shows both FHL and plantar fascia may be involved. The effect of this stretch on the plantar fascia is unclear. Other surgical treatment options for functional hallux rigidus include plantar fascia release, either full or partial, first metatarsal rotational or shortening osteotomies, flexor hallucis brevis and capsular releases, and the aforementioned fibro-osseous tunnel release.

There are different thresholds for the diagnosis of functional hallux rigidus in the literature. Some suggest that 60° of first MTP dorsiflexion is required for normal gait, so it should be used as the cutoff. In our study, the average baseline dorsiflexion was 23.8° and the maximal dorsiflexion was 46° prior to intervention. This would imply that the prevalence of functional hallux rigidus is almost universal. In fact, even after intervention, the maximum dorsiflexion achieved is 58°. Other studies suggest that limitations in dorsiflexion below 60° may be more widespread, but rigid shoewear masks symptoms that would otherwise be problematic [20].

While 60° of first MTP dorsiflexion is optimal for normal gait, as the foot progresses through toe-off, it rises up off the ground, allowing the first metatarsal head to plantarflex. It is unclear how the limitations caused by functional hallux rigidus affect the foot through dynamic motion. As such, 60° may be an unreasonable expectation for closed chain first MTP dorsiflexion. Others in the literature use 20° as the cutoff for functional hallux rigidus [6]. In *Michelson’s* study, all 10 patients with first MTP pain had less than 20° MTP range of motion under simulated weight bearing conditions via the Dananberg technique, but greater than 60° range of motion when the metatarsal and ankle were allowed to plantarflex freely. This is reflected in our average baseline dorsiflexion under simulated closed-chain conditions. Only a single limb of our specimens had baseline dorsiflexion less than 20° and all limbs achieved appropriate dorsiflexion after interventions. Using 20° as the minimum, simulated closed-chain dorsiflexion cutoff will decrease the unintentional misclassification of functional hallux rigidus.

Functional hallux rigidus has been addressed repeatedly in the literature, but there remains significant disagreement regarding the etiology and pathophysiology of this phenomenon. This study only sought to assess the possible contributions of the FHL and plantar fascia. Other possible causes include the flexor hallucis brevis, the first MTP capsule and collateral ligaments, the length of the first metatarsal, the gastrocnemius, and the achilles-calcaneal-plantar system. The lack of statistical significance in this study may be due to one of these other structures being the cause, or the cause may be multifactorial.

There are some limitations to our study. Firstly, because this is a cadaveric study, the specimens are of older age and presumably lower function than the typical patient with functional hallux rigidus. In the largest study of patients the functional hallux rigidus (79 feet in 69 patients), Michelson et al. found the average symptomatic patient was 43 years old and tended to be active, particularly in distance running [18]. While the cadavers were frozen, there may be a difference in the compliance of tissues (including tendinous, fascial, ligamentous, and capsular) as compared to patients. Secondly, while the plantar fascia manipulation was able to be repaired and minimally affected the FHL manipulation, the fibro-osseous tunnel release was performed prior to FHL manipulation and could not be reversed. Also, given the position of the FHL, a reliable repair is not possible, therefore the order of operations could not be randomized. Thirdly, we used the Dananberg technique to asses for first MTP dorsiflexion. Because this is a manual technique, unintentional differences in applied forces may be present. Finally, the small sample size of 14 cadaveric specimen may be underpowered to detect small differences in FHL and PF contributions. Additionally, only the two possible contributors were assessed. Further studies are required to assess other possible contributing structures such as the flexor hallucis brevis and first metatarsal length.

## Conclusion

Functional hallux rigidus causes limitation of first MTP dorsiflexion when the first metatarsal head is prevented from plantarflexing. There is no difference between the contribution of the FHL versus the plantar fascia to functional hallux rigidus. There remain multiple other possible contributing factors to functional hallux rigidus that warrant further study, but the true etiology may be multifactorial. As such, no clear surgical intervention can be recommended to improve functional hallux rigidus. In certain circumstances, releasing the plantar fascia to improve the windlass mechanism or releasing the fibro-osseous tunnel to allow a stenotic FHL may be considered to improve first MTP range of motion.

## Data Availability

No datasets were generated or analysed during the current study.
